# The Impact of a Community-Based Food Education Program on Nutrition-Related Knowledge in Middle-Aged and Older Patients with Type 2 Diabetes: Results of a Pilot Randomized Controlled Trial

**DOI:** 10.3390/ijerph16132403

**Published:** 2019-07-06

**Authors:** Carlos Vasconcelos, António Almeida, Maria Cabral, Elisabete Ramos, Romeu Mendes

**Affiliations:** 1 Department of Sports Sciences, Exercise and Health, University of Trás-os-Montes e Alto Douro, 5000-801 Vila Real, Portugal; 2 Polytechnic Institute of Viseu, 3504-510 Viseu, Portugal; 3 EPIUnit—Instituto de Saúde Pública, Universidade do Porto, 4050-091 Porto, Portugal; 4 Departamento de Ciências da Saúde Pública e Forenses e Educação Médica, Faculdade de Medicina, Universidade do Porto, 4200-319 Porto, Portugal; 5 Public Health Unit, ACES Douro I—Marão e Douro Norte, Northern Region Health Administration, 5000-524 Vila Real, Portugal

**Keywords:** community-based intervention, food education program, nutrition-related knowledge, type 2 diabetes

## Abstract

The purpose of this study was to evaluate the impact of a community-based food education program on nutrition-related knowledge in middle-aged and older patients with type 2 diabetes (T2D). Participants (*n* = 36; 65.9 ± 6.0 years old) were recruited in primary health care to a 9-month community-based lifestyle intervention program for patients with T2D and randomly assigned to an exercise program (control group; *n* = 16) or an exercise program plus a food education program (experimental group; *n* = 20). Nutrition-related knowledge was assessed through a modified version of the General Nutrition Knowledge Questionnaire. The increase in total nutrition-related knowledge score and sources of nutrients area score was significantly higher in the experimental group compared to the control group. No significant changes in nutrition-related knowledge were found between groups in dietary recommendations and diet-disease relationship areas, although improvements were observed. This community-based food education program, with the use of easy to implement strategies (short-duration lectures and dual-task problem solving activities during exercise), had a positive and encouraging impact on nutrition-related knowledge in middle-aged and older patients with T2D.

## 1. Introduction

Diabetes is a public health problem that is increasing worldwide [1]. According to International Diabetes Federation (IDF), in Europe, in 2017, there were 58 million individuals with diabetes [2]. This number is expected to increase by 16% until 2045, reaching the value of 67 million people with diabetes [2]. In Portugal, more than 1 million people between the ages of 20 and 79 have diabetes [3]. Type 2 diabetes (T2D) is the most common type of diabetes [3], and its prevalence is likely to increase with age [4]. The adverse effects of hyperglycemia can be categorized into microvascular (diabetic nephropathy, neuropathy, and retinopathy) and macrovascular complications [5]. Cardiovascular and cerebrovascular diseases are the most common macrovascular complications and the main causes for morbidity and mortality among T2D patients [6]. The adoption of a balanced diet is one of the pillars of diabetes control [7]. For T2D patients, the reduction of energy intake is recommended through the maintenance of a healthy eating pattern based on the intake of unsaturated fats, high fiber foods such as fruits, vegetables, whole grains, and legumes, and limited alcohol intake [2,8]. However, the adherence to nutritional recommendations is generally disregarded by these patients [9]. Nutrition-related knowledge is one of the factors that can influence the adoption of a healthy diet [10] and T2D patients tend to present deficits in this area [11,12,13,14,15]. Therefore it is urgent that this population has access to self-management education focused on nutritional contents. In what concerns diabetes, despite the existence of general nutrition recommendations for self-management education [16], the best intervention remains to be identified [17]. In Portugal, as in many other European countries, caring for people with T2D occurs essentially within the primary health care system [18], which may be a successful place for the implementation of nutritional interventions, due to the fact that there is a closer contact with the patients, and the possibility to provide permanent care [19]. Despite the importance of primary health care, supportive environments are also required to promote behavior changes [20]. Thus, community-based interventions are of particular public health interest as they reach T2D patients in their natural living environment and, when replicated, may attain population level impact [21]. It is the responsibility of nutrition educators to find out innovative solutions so that the community can be aware of the indispensable role of diet in diabetes management [22]. We only found one study in the community setting that analyzed nutrition-related knowledge after the application of a food education program (developed for Korean American Immigrants) [23]. Hence, we carried out a pilot study to evaluate the impact of a community-based food education program on nutrition-related knowledge in middle-aged and older patients with T2D.

## 2. Methods

### 2.1. Study Design

This was a randomized controlled trial for patients with T2D conducted in Vila Real, Portugal, comparing an exercise program only (CON) and an exercise program plus a food education program (EXP). All patients received information to maintain their diabetes management (lifestyle-related physical activity and pharmacological plan), and to continue their medical consultations during the study.

### 2.2. Participants

The implementation of the community-based lifestyle intervention programs was planned for two groups of 25 participants each (the limit of our human, material, and infrastructure resources), representing a total of 274 h of intervention (135 h in the control group and 139 h in the experimental group). Predicting an initial refusal rate of 25%, primary health care medical doctors were asked to select 65 participants according to several inclusion criteria (Table 1).

**Figure 1:**
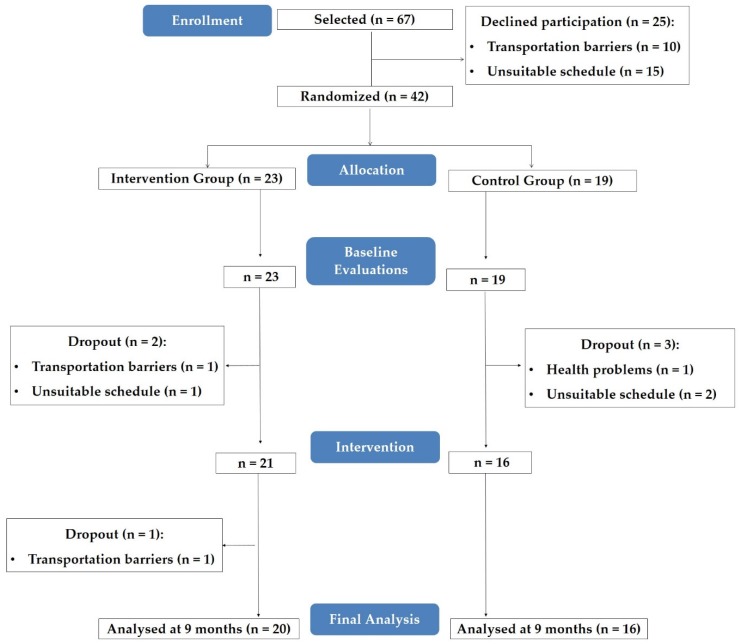
Participants’ flow chart.

Investigators received information of 67 primary health care patients with T2D. A total of 42 agreed to participate in the study and were randomized, following simple randomization procedures with the use of a computer software, for CON (19 patients) or EXP (23 patients) (Figure 1). 

Level of adherence to the food education program was not considered an exclusion criterion for final analysis. The reasons for dropouts are explained in Section 3.

The study protocol was approved by the local health ethics committee in accordance with the Declaration of Helsinki. All patients gave written informed consent before participation, including capture images. The trial was registered at ClinicalTrials.gov, number NCT02631902.

### 2.3. Exercise Program

All patients participated in a 9-month community-based exercise program [24,25], developed according with international exercise recommendations for patients with T2D [26]. As the mean age of our participants was 65.9 ± 6.0 years old, the exercise program was also based in international recommendations on falls prevention [27]. This program consisted of three exercise sessions per week, 75 min per session, combining aerobic, resistance, agility/balance, and flexibility exercise.

### 2.4. Food Education Program

**Figure 2:**
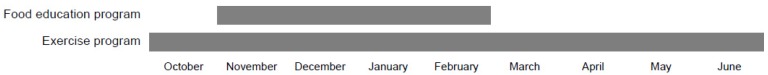
Food education program and exercise program timeline.

**Figure 3:**
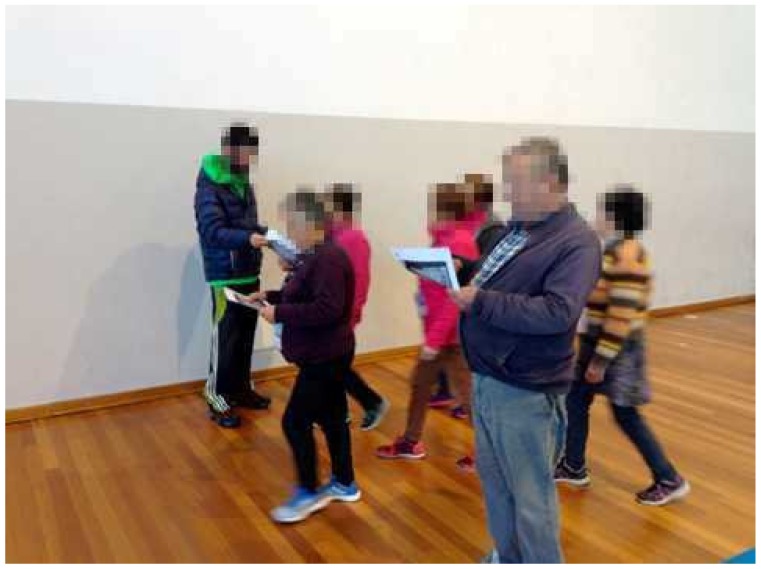
Food label interpretation during walking exercise. Participants were asked to select, among two, which food label had more carbohydrate, added sugar, fat, or saturated fat.

**Figure 4:**
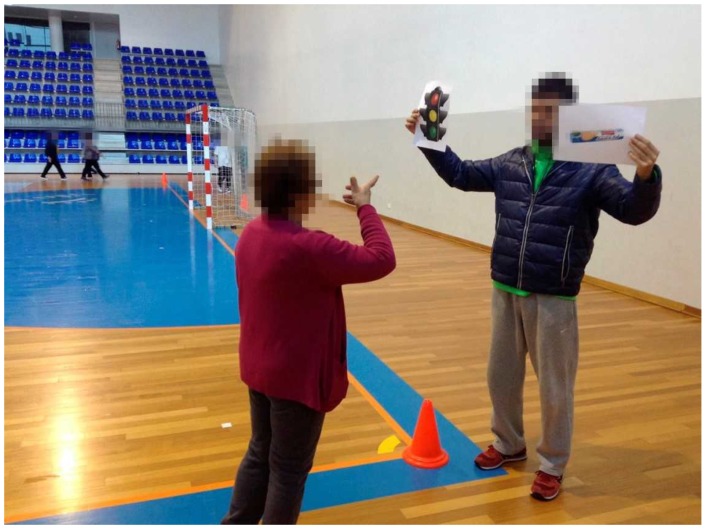
Traffic light system (individual response) during walking exercise: green color—best food choices; yellow color—choose carefully; red color—foods to avoid.

**Figure 5:**
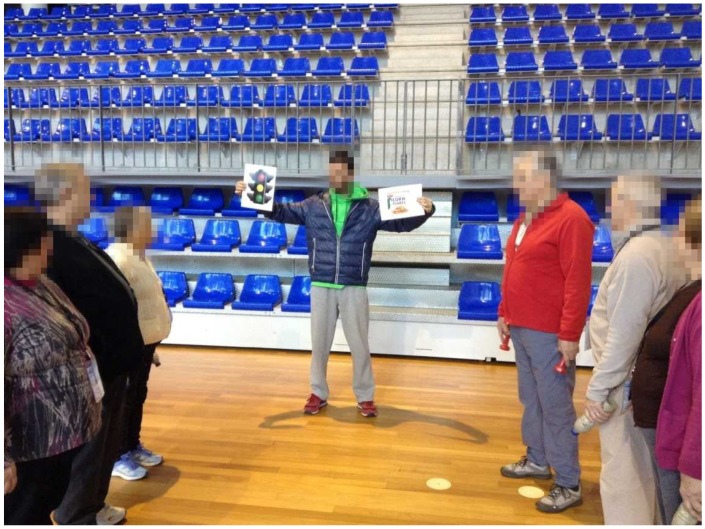
Traffic light system (group discussion) during walking exercise: green color—best food choices; yellow color—choose carefully; red color—foods to avoid.

**Figure 6:**
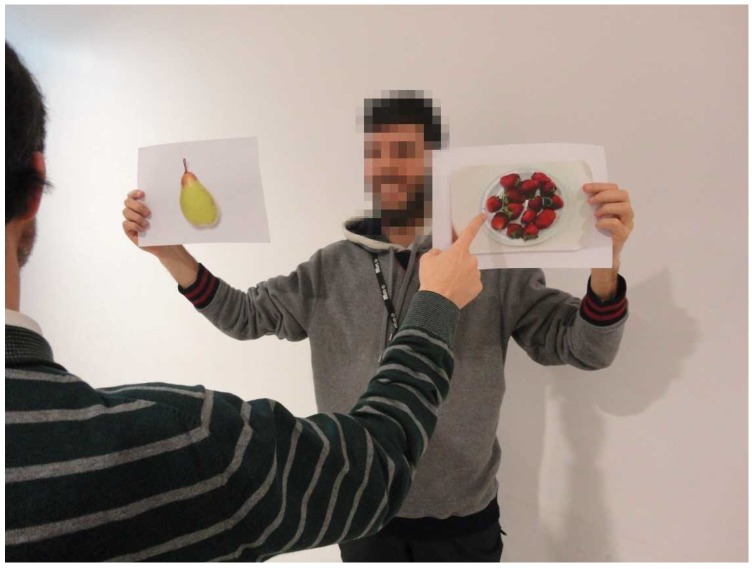
Multiple choice answer during walking exercise. Participants were asked to select, among two, which foods had more sugar, fat, saturated fat, glycemic index, or glycemic load.

**Figure 7:**
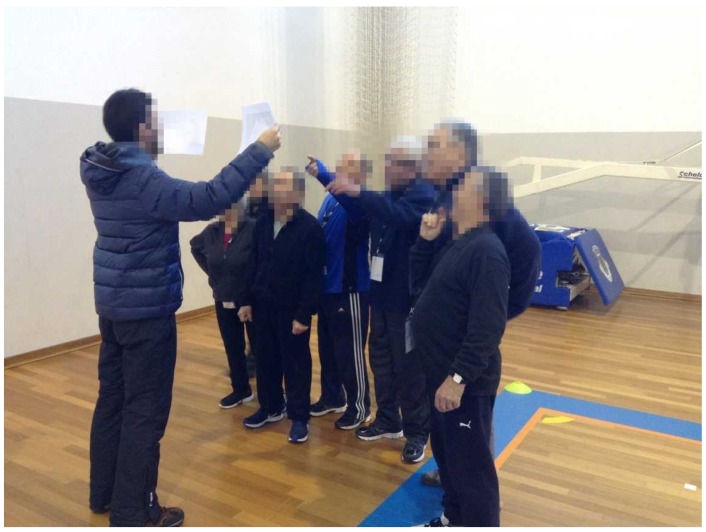
Multiple choice answer (group discussion) during walking exercise. Participants were asked to select, among two, which foods had more sugar, fat, saturated fat, glycemic index, or glycemic load.

Patients randomized to the EXP group received, plus the exercise program, a 16-week food education program (Figure 2) based on IDF nutrition teaching modules [28], and American Diabetes Association (ADA) dietary recommendations for T2D management [8]. On each week, a different nutrition-related content (Table 2) was addressed through two sessions: (1) a theoretical session of 15 min performed before one exercise session; and (2) dual-task strategies integrated in another exercise session: (food label interpretation (Figure 3); traffic light system with individual response (Figure 4) or group discussion (Figure 5); multiple choice answers after one question with individual response (Figure 6), or group discussion (Figure 7)). Attendance was recorded at each session.

### 2.5. Evaluations

Demographic data, such as age, gender, education level, personal monthly (net) income, marital status, and living situation were recorded by a standard questionnaire. Diabetes data, such as HbA1c and diabetes duration were collected by primary health care medical doctors. HbA1c was assessed by a fasting (minimum of 8 h) venous blood analysis according to standard international laboratory methods before entrance in the study. Cognitive function was also evaluated by the investigators through Mini Mental State Examination, with all patients included regardless of classification.

Nutrition-related knowledge was assessed, before and after the 9-month intervention, by the Portuguese modified short version [29] of the General Nutrition Knowledge Questionnaire (GNKQ) [30]. This version of the questionnaire consisted of three sections: dietary recommendations (DR, 0–6 points); sources of nutrients (SN, 0–34 points), and diet-disease relationship (DDR, 0–16 points). Correct responses from each section were added, giving an overall score out of 56 points. Differences between the original questionnaire and the one used in our study are presented in Table 3. Participants answered on a range of different scales, such as ‘more, equal, less, don’t know’, ‘yes, no, don’t know’, ‘rich, poor, don´t know’, ‘agree, disagree, don´t know’. In the diet-disease relationship section, some items were open-ended and required participants to list diseases associated with diet-related lifestyle factors.

### 2.6. Data Analysis

Data are shown as mean ± SD for continuous variables and as proportions (number and percentage) for categorical variables.

To compare the effects of the time * group interaction on nutrition-related knowledge, analysis of variance (ANOVA) with repeated measures was performed. Partial eta squared values (µ^2^_p_) were reported to quantify the effect sizes. To identify factors that were independently associated with significant improvements in nutrition-related knowledge in the EXP group, a multiple linear regression analysis was performed. The level of statistical significance was set at *p* < 0.05 and data were analyzed with PASW Statistics version 20 (IBM SPSS, Hong Kong, China).

## 3. Results

### 3.1. Program Implementation

From the individuals initially selected, 37% refused to participate in the study, indicating as reasons transportation barriers or unsuitable schedule. Thus, 42 participants were randomized and evaluated (23 in the EXP group and 19 in the CON group). Prior to the start of the intervention, five participants dropped out (two in the EXP group (unsuitable schedule, *n* = 1, transportation barriers, *n* = 1) and three in the CON group (health problems, *n* = 1, unsuitable schedule, *n* = 2). Another dropout in EXP group was verified during the intervention due to transportation barriers (*n* = 1).

In the final analysis, 16 patients from the CON group and 20 patients from the EXP group were included (Figure 1). Attendance to the food education program was 47.5% ± 27.1% (ranging from 2.9% to 85.3%).

### 3.2. Participants’ Characteristics

The mean age of the 36 participants was 65.9 ± 6.0 years old (20 males). Participants had T2D diagnosed at 6.3 ± 5.2 years and HbA1c of 7.0% ± 1.1%. Final sample characteristics are presented in Table 4.

### 3.3. Nutrition-Related Knowledge

The mean values of nutrition-related knowledge (total and per section) in CON and EXP groups in the two evaluation moments are presented in Table 5. The increase in total nutrition-related knowledge and SN area was significantly higher in the EXP group compared to the CON group. No significant changes in nutrition-related knowledge were found between groups in DR and DDR areas, although improvements were observed, mainly in the EXP group.

Table 6 shows the factors independently associated with the increase in nutrition-related knowledge in the EXP group. After adjustment, increase in nutrition-related knowledge was significantly and negatively associated with diabetes duration (β = −0.543, *p* = 0.020) and significantly and positively associated with attendance to the food education program (β = 0.096, *p* = 0.011).

Participants performed a Mini Mental State Examination to evaluate cognitive function. According to cutoffs from the Portuguese adapted version of this instrument [31], five patients (three from CON and two from EXP groups) were classified with cognitive impairment. For ethical reasons, these individuals were not excluded from participation in the study neither from the final analysis. In these patients, nutrition-related knowledge between the baseline assessment and the final analysis increased 8.3% (1.2% in the CON group and 19.6% in the EXP group).

## 4. Discussion

Our study revealed that a community-based food education program significantly increased nutrition-related knowledge in middle-aged and older patients with T2D. Participants’ global improvements are mostly derived from the evolution in scores of sources of nutrients. The emphasis of our food education program on contents from this knowledge area is likely to be the reason for this change. On the contrary, differences between groups were not found in dietary recommendations and diet-disease relationship areas. In dietary recommendations, participants from CON and EXP groups started with a high score (5.0 and 4.9 points respectively) in a maximum section scale of 6 points, which makes improvements harder to achieve. Regarding the diet-disease relationship area, the results may have been conditioned by the design of the questions, as half of questions were open-ended [32]. Furthermore, this was the final section of the instrument and, according to Rolstad et al. [33], the length of the survey can increase response burden.

The increase of nutrition-related knowledge is associated with healthier food patterns [10] and may lead to a better glycemic control in T2D patients [34]. Nutrition-related knowledge is a key component of diabetes knowledge. Most educational interventions analyzed in scientific studies with T2D patients focused on diabetes knowledge without presenting results for patients’ knowledge about nutrition management of diabetes [35,36,37,38,39]. Some studies showed the effectiveness of a nutritional intervention in nutrition-related knowledge of T2D patients [22,40,41,42,43]. However, as far as we know, there is only one published RCT conducted in a community setting, with Korean American immigrants [22], that assessed the effectiveness of a nutrition education program on nutrition-related knowledge in patients with T2D (*n* = 79; 56.5 ± 7.9 years). After two group face-to-face sessions (two hours each; one week apart) devoted to nutritional contents, there were significant differences in nutrition-related knowledge with the intervention group scoring better, as assessed after 30 weeks.

It is difficult to discuss between our study and that of Song et al. [22] because of different sample characteristics, assessment instruments, and baseline scores. As explicit memory (recognition) declines with age, the fact that average age of our participants was higher than Song’s study (65.9 ± 6.0 vs. 56.5 ± 7.9 years) makes their learning more difficult [44]. Song et al. [22] used the Diabetes Knowledge Test with seven multiple choice questions for nutrition-related knowledge, while in our trial we applied the GNKQ, comprised of three different areas of nutrition-related knowledge (DR, SN, and DDR). According to Worsley [45], it is of crucial importance to measure different areas of nutrition-related knowledge, as this outcome is not one dimensional. Contrary to Song’s results, participants from our trial started with a baseline score above 50% (29.8 out of 56 points), being more difficult to improve nutrition-related knowledge.

In our study, we used face-to-face group education delivered over 16 weeks, lasting a total of 12 h. The classes were based on IDF nutrition teaching modules [27] and ADA dietary recommendations for T2D control [28], given through theoretical classes together with dual-task problem solving strategies during exercise.

Face-to-face education is one of the most common educational methods, as it enables patients to ask and discuss their doubts, allowing the construction of a dynamic relationship between the educator and patients [46]. Despite this, there are a rising number of studies conducted in T2D patients delivering education through technology-based methods [35,47,48,49,50]. Although technology-based programs have the potential to solve the problem of distance to the place of intervention, they can represent a barrier to patients from lower socioeconomic and educational groups, such as the patients from our EXP group, who are more likely to have lower digital literacy and more difficulties in the access and use of technologies [51].

According to Coppola et al. [52], there are three different methods to provide patient education: during usual care, structured group, and individual education. Two meta-analyses [53,54] showed the benefits of group-based education on diabetes knowledge, when compared with individual education. Group education provides opportunities for patients’ interactions, making possible discussions about several topics. Moreover, it provides support from others facing similar challenges, allowing participants to feel integrated in a group context [55].

In what concerns the duration of nutritional programs, interventions conducted in T2D patients presented variable results, ranging from 2 h and 40 min [56] to 25 h [42] and being delivered between 1 week [22,49] to 6 months [43,57]. Steinsbeck et al. [54], in a systematic review of group-based T2D self-management education, concluded that interventions delivered between 6 and 10 months and with 19 to 52 h of duration give the best results in diabetes knowledge.

Regarding the development of food education programs, ADA recommendations for T2D management were also used by Song et al. [22], with some contents similar to those used in our study, such as carbohydrate counting, food pyramid, healthy eating plate, and meal planning. Other nutritional interventions that improved diabetes knowledge in T2D patients also had similar contents to our study: definition of diabetes [36,37,38], meal planning [38], meal frequency [38], cooking methods [37], importance of fruit, vegetable, and whole grains [37], and healthy eating [36,38].

Our intervention was centered on two teaching methods: structured lecture (15-min group class) and dual-task problem solving (30 min integrated in one exercise session). Lectures are the best teaching method to transmit declarative knowledge [58]. Problem solving tasks are a great indicator of functional ability in the elderly [59]. Furthermore, dual-task problem solving—in this study the completion of a secondary task while walking—is a key contributor for the prevention of falls in the elderly [60]. The use of this technique during exercise was the most innovative aspect of our study. In addition to preparing our patients to the dual-task problem solving of the daily life, it also allows them to target the lifestyle factors of T2D management. Although cognitive impairment may affect learning behaviors [61], the evolution of nutrition-related knowledge, even in these patients (assessed with Mini Mental State Examination), proves the efficacy of these simple teaching methods.

As expected, attendance to food education program was an independent factor associated with the increase of nutrition-related knowledge in our EXP group. As in our study, Bruce et al. [62] and Brown et al. [63] also found that higher attendance to educational sessions was related to greater knowledge levels.

Attendance to our food education program presented lower values compared with other educational interventions in T2D patients (47.5% vs. 72.5% [64], 74% [65], and 78% [66]). Attendance to interventions has a natural influence on its efficacy [67]. Identification of the motives of low attendance rates is of crucial importance for food education program feedback [68]. In our study, participants’ attendance was tracked. Whenever an individual missed two sessions in a row, a phone call was made to record the cause. Patients reported health status, weather, work, family activities, and transportation as constraints, meeting the reasons listed by Brzoska and Misra [69]. Therefore, there is a need for strategies to increase attendance to education sessions in people with T2D. Miller et al. [41,42] also tracked participant’s attendance. If T2D patients missed a group session, they were stimulated to attend a backup session. Participation of family members in the education sessions [22,70] and the presence of the community health professionals [22,71] are two other strategies that were used in interventions with T2D patients to promote class attendance.

The negative association between diabetes duration and the evolution in nutrition-related knowledge is another finding from our study. Hassing et al. [72] reported an association between diabetes mellitus and mild cognitive impairment, which is higher with longer duration of diabetes [73]. The accurate pathophysiology of cognitive dysfunction in diabetes is not totally defined, but probably hyperglycemia, vascular disease, hypoglycemia events, and insulin resistance are the main factors [74]. Diabetes is typically a progressive chronic disease that is often related to emotionally stressful events [75]. Chronic stress affects the function of the cognitive system, having implications for educational contexts [76]. According to Eom et al. [77], individuals with more years of diabetes have more diabetes-related stress.

The present study has some limitations that need to be addressed. Our food education program had an average low attendance level (48%). Another factor that should be highlighted was the existence of 37% of declines to participation from those originally selected by their medical doctors. Besides, we had 16% of dropouts following randomization. This underlines the difficulty in implementing lifestyle interventions for this population, even when culturally adapted and free of charge. Despite randomization, there is a five-year difference in the mean of the age between CON and EXP groups, quite a large difference for such a small trial.

Our study was strengthened by its randomized controlled design, the control of the effects of covariates on the evolution of nutrition-related knowledge in the EXP group, the application of short-duration lectures and the use of an innovative method (dual-task strategy) to give nutritional contents. Moreover, our questionnaire allowed data collection from three different areas of nutrition-related knowledge. In accordance to our search, this was the first study conducted in Portugal that evaluated the effect of a community-based food education program in nutrition-related knowledge, in middle-aged and older patients with T2D, in primary health care. Diabetes is a major public health problem in Portugal and worldwide.

## 5. Conclusions

In conclusion, this community-based food education program, with the use of easy to implement strategies (short-duration lectures and dual-task problem solving activities during exercise), had a positive and encouraging impact on nutrition-related knowledge in middle-aged and older patients with T2D. More research should be done to understand to which extent this increase in nutrition-related knowledge has an impact on dietary intake and diabetes-related outcomes.

## Figures and Tables

**Table 1:** Inclusion criteria for participation in the study.

Inclusion Criteria for Participation in the Study
Diagnosed with T2D for at least six months
Aged between 50 and 80 years old
Non-smokers
Not engaged in supervised exercise
Independent living in the community
Medical recommendation for lifestyle intervention
Known medical history
Diabetes comorbidities under control (diabetic foot, retinopathy, and nephropathy)
No cardiovascular, respiratory, and musculoskeletal contraindications to exercise
Without major problems in gait or balance
Not started insulin therapy in the past three months

**Table 2:** Contents of the food education program.

Week	Contents
Week 1	Diabetes, insulin, and glycaemia
Week 2	Functions of nutrients
Week 3	Sources of nutrients
Week 4	Food Wheel (fruit, vegetables, cereals, rice, and potatoes)
Week 5	Food Wheel (meat, fish and eggs, dairy products, fats, and oils)
Week 6	Glycemic index and glycemic load
Week 7	Added sugars
Week 8	Carbohydrate counting
Week 9	Food label interpretation (carbohydrates; sugars)
Week 10	Food label interpretation (fats; saturated fats)
Week 11	Dietetic products (lean, diet, light, zero)
Week 12	Fats
Week 13	Soup and salt
Week 14	Drinks
Week 15	Cooking methods
Week 16	Meal planning and the healthy eating plate

**Table 3:** Differences between the General Nutrition Knowledge Questionnaire and the Portuguese modified short version of the General Nutrition Knowledge Questionnaire.

Section	GNKQ	Portuguese Modified Short Version of GNKQ
Section 1	Three multiple choice and one open-ended question	One multiple choice question
Section 2	21 multiple choice questions	Eight multiple choice questions
Section 3	10 multiple choice questions	No questions—lack of internal consistency and item validation
Section 4	Five multiple choice and five open-ended questions	Five multiple choice and four open-ended questions

GNKQ: General Nutrition Knowledge Questionnaire.

**Table 4:** Characteristics of the study participants according to group.

Characteristics	CON Group (*n* = 16)	EXP Group (*n* = 20)
Age, mean ± SD	63.00 ± 5.39	68.25 ± 5.60
Gender, *n* (%)		
Male	10 (62.5)	10 (50.0)
Female	6 (37.5)	10 (50.0)
Education level, *n* (%)		
≤4 years	9 (56.3)	11 (55.0)
5 to 9 years	4 (25.0)	6 (30.0)
>9 years	3 (18.8)	3 (15.0)
Personal monthly income, *n* (%)		
<500 €	3 (18.8)	11 (55.0)
Between 500 and 1000 €	7 (43.7)	6 (30.0)
More than 1000 €	6 (37.5)	3 (15.0)
Marital status, *n* (%)		
Single, divorced, or widower	3 (18.8)	4 (20.0)
Married or with domestic partner	13 (81.2)	16 (80.0)
Living situation, *n* (%)		
Living alone	1 (6.3)	3 (15.0)
Living with others	15 (93.7)	17 (85.0)
Glycated hemoglobin, mean ± SD	6.87 ± 1.13	7.18 ± 1.13
Diabetes duration, mean ± SD	7.63 ± 5.73	5.30 ± 4.57
Mini Mental State score, mean ± SD	26.56 ± 2.94	26.25 ± 2.97

CON: Control; EXP: Experimental.

**Table 5:** Nutrition-related knowledge (total and per section) in two evaluation moments in both groups.

Nutrition-Related Knowledge (Points)	Control Group		Experimental Group		*p*	η^2^_p_
	Baseline	9 Months	Baseline	9 Months		
Total score	30.2 ± 6.1	31.3 ± 7.4	29.4 ± 6.2	35.2 ± 5.7	0.001	0.290
Dietary recommendations section	5.0 ± 0.5	5.1 ± 0.6	4.9 ± 0.8	5.5 ± 0.6	0.053	0.106
Sources of nutrients section	18.8 ± 4.9	19.1 ± 5.5	17.8 ± 5.1	21.9 ± 4.5	0.004	0.217
Diet-disease relationship section	6.4 ± 1.5	7.1 ± 2.1	6.8 ± 1.7	7.9 ± 1.5	0.513	0.013

*p*: *p*-value of the time * group interaction effect determined by analysis of variance with repeated measures; η^2^_p_: partial eta squared.

**Table 6:** Factors independently associated with the increase in nutrition-related knowledge in the experimental group.

Factors	β (95% CI)	*p*
Age	−0.043 (−0.355, 0.270)	0.763
Gender	0.710 (−2.814, 4.234)	0.659
Education level	−2.819 (−6.015, 0.378)	0.077
Personal monthly income	1.522 (−1.632, 4.677)	0.303
Marital status	4.034 (−2.649, 10.717)	0.205
Living situation	−3.394 (−10.682, 3.895)	0.320
Glycated hemoglobin	0.154 (−1.504, 1.811)	0.839
Diabetes duration	−0.543 (−0.977, −0.109)	0.020
Mini Mental State Examination score	−0.042 (−0.863, 0.778)	0.910
Attendance to food education program	0.096 (0.028, 0.165)	0.011

β: standard coefficient determined by multiple regression analysis; CI: confidence interval; *p*: *p*-value of the association of independent factors with nutrition-related knowledge in the experimental group.
